# Supplementary material for: The emerging role of drought as a regulator of dissolved organic carbon in boreal landscapes
Source: Nat Commun. 2022 Aug 31;13:5125. doi: 10.1038/s41467-022-32839-3 (PMC9433396; doi:10.1038/s41467-022-32839-3)
Supplement: Supplementary file 1 — Supplementary Information [file 41467_2022_32839_MOESM1_ESM.pdf]

Supplementary information for “The emerging role of drought as a regulator of dissolved organic carbon in boreal landscapes”

***Tejshree Tiwari[1], Ryan A. Sponseller[2], and Hjalmar Laudon[1].**

[1]Department of Forest Ecology and Management, Swedish University of Agricultural Sciences, SE-901 83 Umea, Sweden

[2]Department of Ecology and Environmental Sciences, Umea University, 901 87 Umea, Sweden.

Supplementary Figures

Contents of this file

Supplementary Figure 1

Supplementary Figure 2

Supplementary Figure 3

Supplementary Figure 4

Supplementary Table 1

Supplementary Table 2

Introduction

This document provides four supporting figures and two tables that contribute additional information to the findings presented in this manuscript.

Figures

Supplementary Fig. 1 Drought and post-dought effects were similar whether or not DOC was normalized for discharge at the time of sampling. **a** and **b** shows drought and post-drought effects using normalized dissolved organic carbon (DOC) and plotted against the number of summer low flow days during drought (a) and after to rewetting (b) (drought r^2 range 0.31-0.68, $p<0.05$ and post-drought r^2 range 0.20-0.57, $p<0.05$). The plots in **c** and **d** are uncorrected DOC concentrations also plotted against summer low flow day as in panels a and b (drought r^2 range 0.29-0.65, $p<0.05$ and post-drought r^2 range 0.28-0.67, $p<0.05$).

Supplementary Fig. 2 Dissolved organic carbon (DOC) exports (% change) in relation to summer low flows for each monitoring site in the Krycklan catchment. The top panel provides the export estimates only for the summer (June-August); the middle panel provides estimates during the autumn (October-November); the bottom panel provides annual estimates.

Supplementary Fig. 3 Drought and post-drought effects on groundwater DOC responses to summer drought severity. The responses for dissolved organic carbon (a, b), low molecular weight DOC (LMW DOC) (c, d), carbon to nitrogen ratio (C/N ratio) (e, f), and specific UV absorbance at 254 nm ($SUVA_{254}$) (g, h). Forest values represent averages from lysimeter samples collected between 0.1 and 0.65 m depth in the riparian zone, while values from the mire wells represent averages from samples collected at 2-2.5 m. In both cases, this depth range corresponds to the location of dominant hydrologic flow paths that move through these patches [1].

Supplementary Fig 4. DOC slope responses to summer low flows, modeled using the best predictor (catchment size) during drought and post-drought. Note that only values from significant regression lines were used in the models. Note also that these values are relative changes in relation to an individual catchment's long-term averages and pre-drought conditions rather than absolute changes in DOC concentrations.

Tables

Supplementary Table 1. Drainage size (km^2) and percent coverage of forest and peatlands within the 13 catchments used in this study.

Supplementary Table 2. Average summer values of dissolved organic carbon (DOC) concentration, the low molecular weight DOC (LMW DOC), the carbon to nitrogen ratio (C/N ratio), and specific UV absorbance at 254 nm ($SUVA_{254}$)

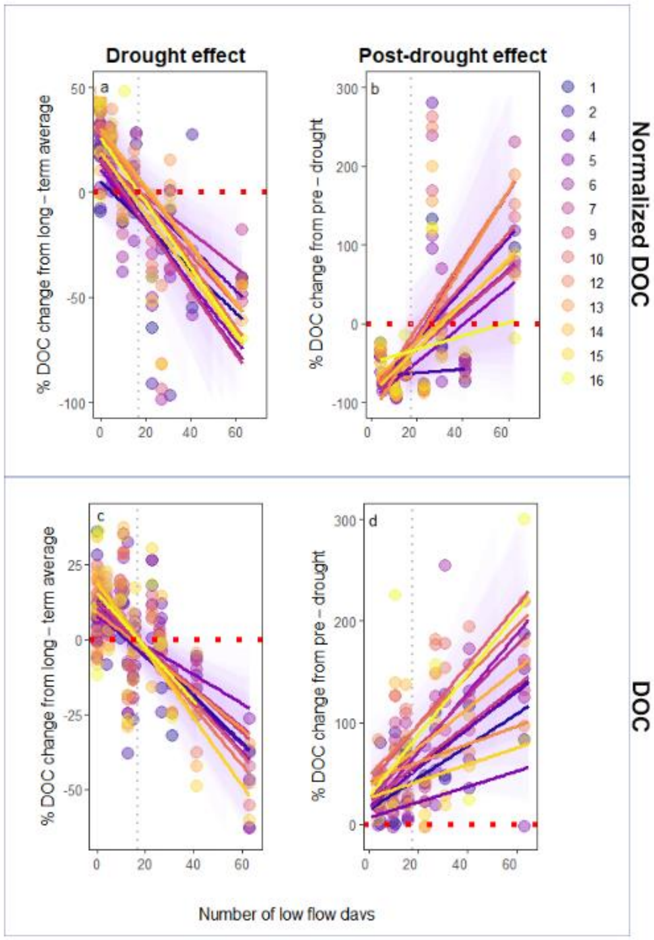

Supplementary Fig. 1 Drought and post-dought effects were similar whether or not DOC was normalized for discharge at the time of sampling. **a** and **b** shows drought and post-drought effects using normalized dissolved organic carbon (DOC) and plotted against the number of summer low flow days during drought (a) and after to rewetting (b) (drought r^2 range 0.31-0.68, $p<0.05$ and post-drought r^2 range 0.20-0.57, $p<0.05$). The plots in **c** and **d** are uncorrected

DOC concentrations also plotted against summer low flow day as in panels a and b (drought r^2 range 0.29-0.65, $p<0.05$ and post-drought r^2 range 0.28-0.67, $p<0.05$).

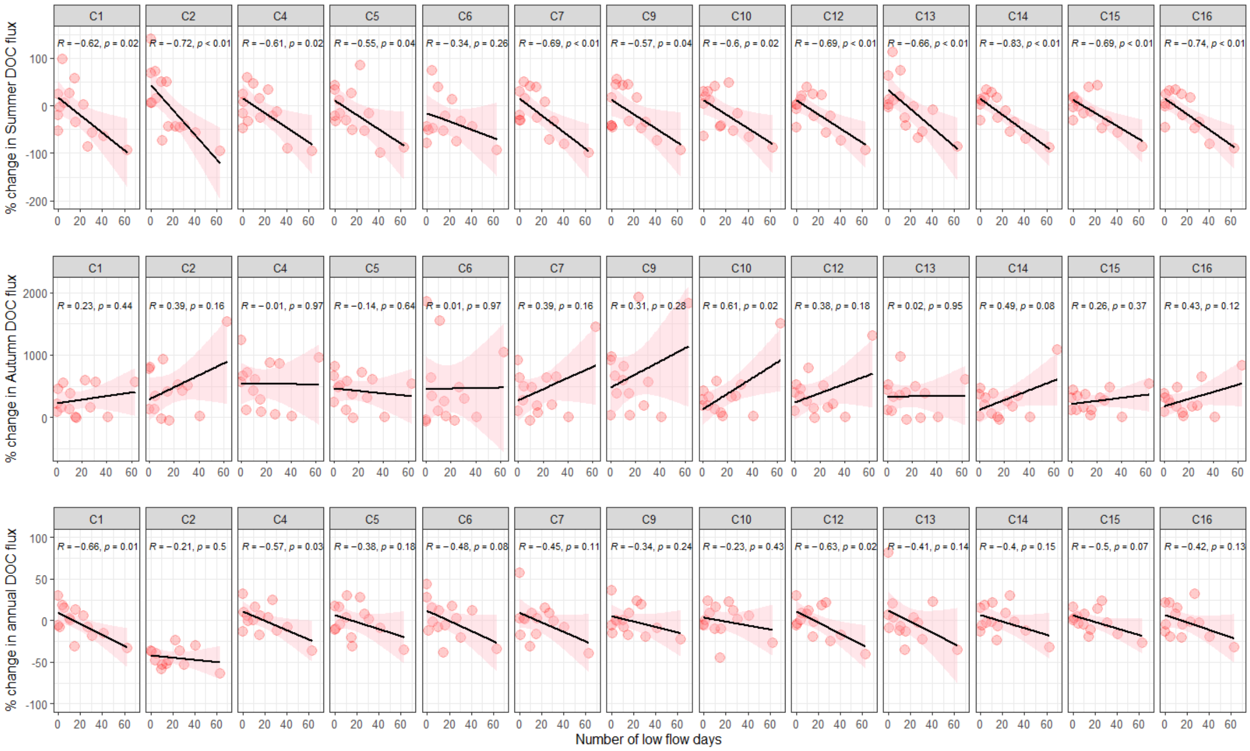

Supplementary Fig. 2 Dissolved organic carbon (DOC) exports (% change) in relation to summer low flows for each monitoring site in the Krycklan catchment. The top panel provides the export estimates only for the summer (June-August); the middle panel provides estimates during the autumn (October-November); the bottom panel provides annual estimates.

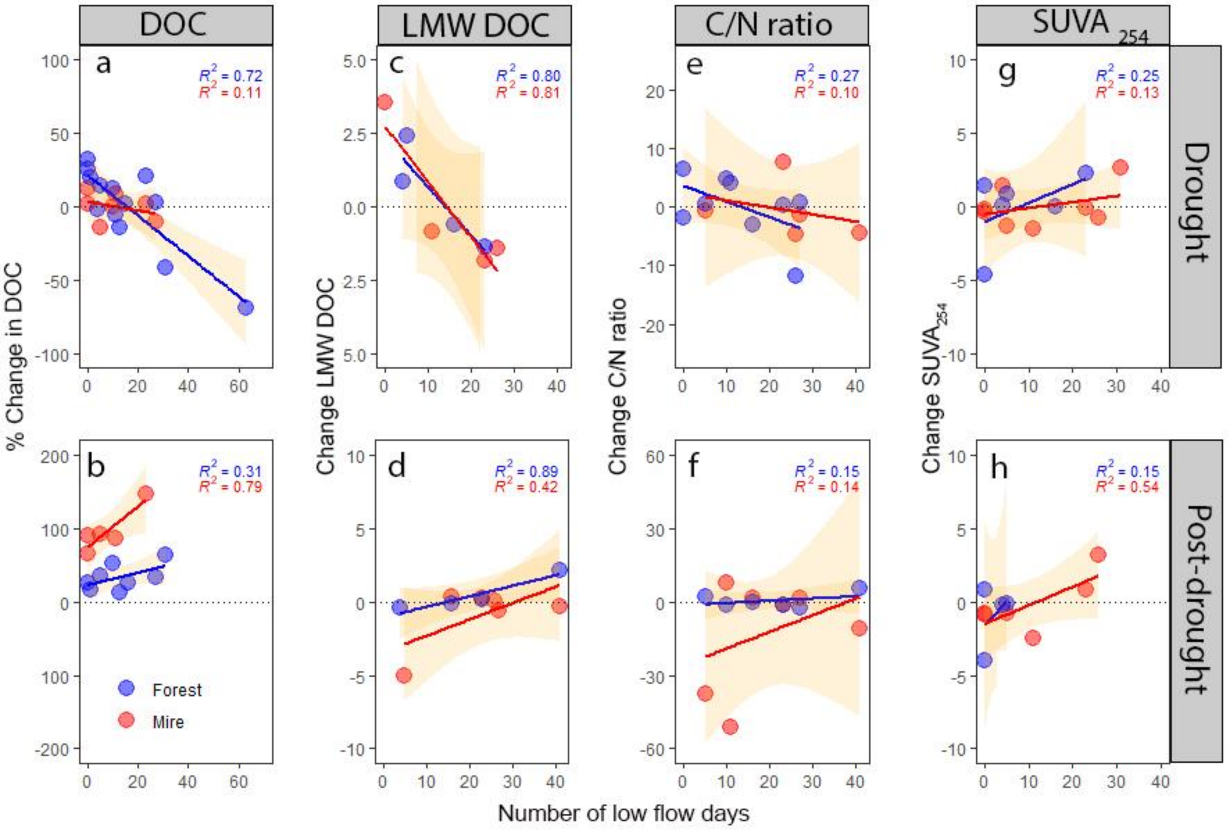

Supplementary Fig. 3 Drought and post-drought effects on groundwater DOC responses to summer drought severity. The responses for dissolved organic carbon (**a, b**), low molecular weight DOC (LMW DOC) (c, d), carbon to nitrogen ratio (C/N ratio) (**e, f**), and specific UV absorbance at 254 nm ($SUVA_{254}$) (g, h). Forest values represent averages from lysimeter samples collected between 0.1 and 0.65 m depth in the riparian zone, while values from the mire wells represent averages from samples collected at 2-2.5 m. In both cases, this depth range corresponds to the location of dominant hydrologic flow paths that move through these patches [1].

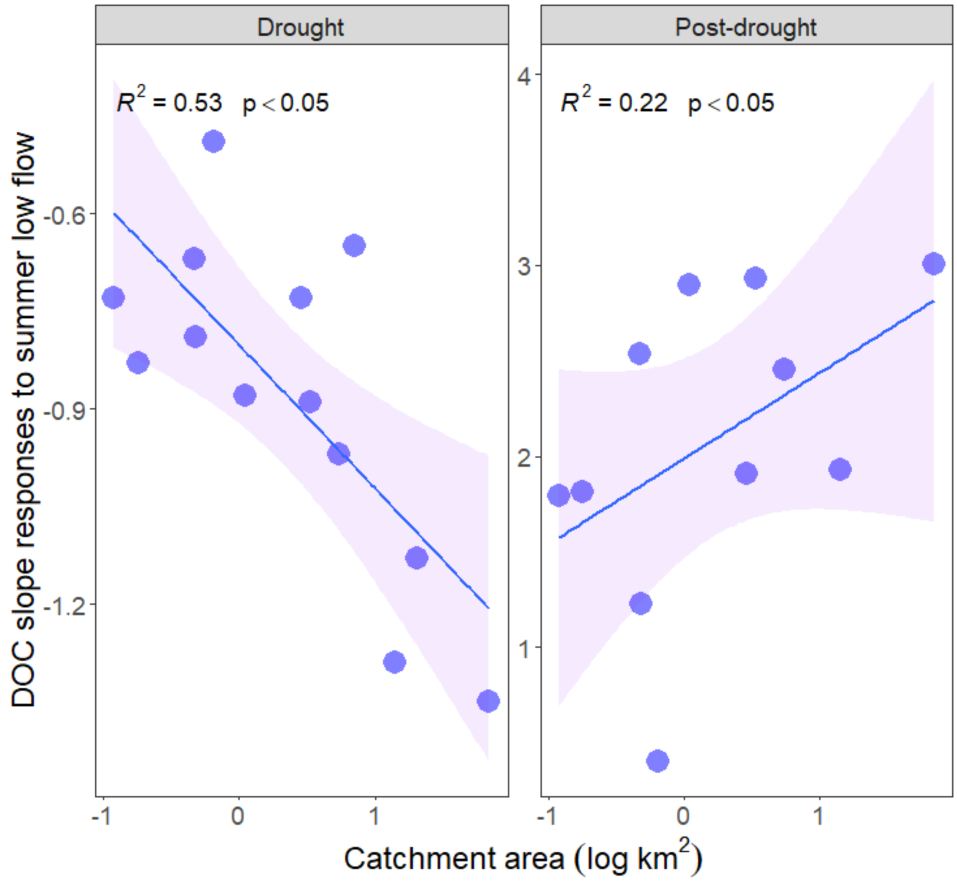

Supplementary Fig 4. DOC slope responses to summer low flows, modeled using the best predictor (catchment size) during drought and post-drought. Note that only values from significant regression lines were used in the models. Note also that these values are relative changes in relation to an individual catchment's long-term averages and pre-drought conditions rather than absolute changes in DOC concentrations.

Supplementary Table 1. Drainage size (km^2) and percent coverage of forest and peatlands within the 13 catchments used in this study.

Catchments	Area (km^2)	Peat (%)	Forest (%)
1	0.48	0.00	97.95
2	0.12	0.00	99.92
4	0.18	51.06	55.88
5	0.65	47.76	53.98
6	1.1	28.66	71.36
7	0.47	19.41	81.95
9	2.88	15.16	84.36
10	3.36	28.84	73.84
12	5.44	19.16	82.57
13	7	11.81	88.19
14	14.1	6.64	90.13
15	20.13	14.50	81.57
16	67	9.40	87.20

Supplementary Table 2. Average summer values of dissolved organic carbon (DOC) concentration, the low molecular weight DOC (LMW DOC), the carbon to nitrogen ratio (C/N ratio), and specific UV absorbance at 254 nm ($SUVA_{254}$)

Sites	DOC (mg L^{-1})	LMW DOC	C/N ratio	$SUVA_{254}$
C1	21.4	4.6	47.8	4.4
C2	21.4	4.4	53.8	4.3
C4	38.0	3.9	65.6	4.6
C5	17.9	3.8	45.4	5.1
C6	16.5	3.9	48.1	4.9
C7	27.4	3.9	57.1	5.0
C9	17.1	4.1	48.9	5.0
C10	22.8	3.9	57.0	4.5
C12	21.1	4.0	55.4	4.3
C13	22.3	4.7	52.8	4.9
C14	13.3	4.3	42.8	5.1
C15	11.6	4.1	43.5	4.7
C16	10.6	4.1	40.2	4.7

Supplementary Reference

1 Laudon, H. *et al.* The Krycklan Catchment Study-A flagship infrastructure for hydrology, biogeochemistry, and climate research in the boreal landscape. *Water Resour Res* **49**, 7154-7158, doi:10.1002/Wrcr.20520 (2013).
